# Supplementary material for: The effect of melatonin supplementation on the plasma levels of 2-arachidonoylglycerol, ghrelin and hedonic eating intensity in overweight/obese females: A study protocol for a pilot randomized controlled trial
Source: PLoS One. 2025 Apr 22;20(4):e0319258. doi: 10.1371/journal.pone.0319258 (PMC12013906; doi:10.1371/journal.pone.0319258)
Supplement: S1 File — (DOCX) [file pone.0319258.s002.docx]

بسمه تعالی

برگ نخست

حاوی اطلاعات براي مشاركت كننده تاريخ : 04/10/1401

**عنوان/موضوع تحقيق: بررسی اثر مکمل یاری ملاتونین بر سطوح پ****لاسمایی گرلین، 2-آراشیدونیل گلیسرول و شدت خوردن هدونیک**

**نوع تحقيق: این مطالعه از نوع کارآزمایی بالینی تصادفی کنترل دار دوسوکور است.**

**خانم محترم**

از شما دعوت مي شود در يك مطالعه تحقيقاتي كه توسط اساتید دانشکده ی تغذیه و علوم غذایی دانشگاه علوم پزشکی تبریز تحت نظارت کميته منطقه اي اخلاق دانشگاه انجام يافته و توسط معاونت تحقیقات و فناوری دانشگاه علوم پزشکی تبریز تأمین مالی می شود شركت كنيد. پيش از آنكه تصميم به شركت ياعدم شركت بنمائيد، من تحقيق را بطور خلاصه براي شما توضيح مي دهم: چرا اين تحقيق صورت مي گيرد و اين تحقيق مستلزم چه چيزي است. لطفا" در خواندن اطلاعات زير در مورد تحقيق عجله نكنيد و آنها را بدقت بخوانيد. هر كجا نياز به توضيح داشتيد سوال فرمائيد و در باره تصميم در مورد شركت يا عدم شركت خودتان عجله نكنيد. **هزینه ای از بابت پژوهش اخذ نخواهد شد. همچنین در صورتی که به دلیل هر نوع بیماری تحت درمان هستید در طی مطالعه از درمان روتین محروم نخواهید شد.**

**هدف اين مطالعه (تحقيق) چيست؟** **و چگونه انجام خواهد شد؟ (هدف و روش)**

کنترل چاقی نقش موثری در پیشگیری از بیماری ها دارد. یکی از علل مهم چاقی و اضافه وزن، خوردن هدونیک یعنی خوردن غیر ارادی و خارج از کنترل فرد می باشد که بدون توجه به نیاز بدن عمل خوردن صورت می پذیرد و کنترل خوردن هدونیک اهمیت زیادی در کنترل چاقی دارد. هدف این تحقیق یافتن راهکاری در جهت کنترل خوردن هدونیک است. طبق تحقیقات اخیر مکمل بدون عارضه ملاتونین علاوه بر نقشی که در تنظیم خواب دارد، به نظر می رسد می تواند بر خوردن هدونیک و کاهش وزن نیز موثر باشد. در این تحقیق اثر مکملیاری ملاتونین بر وضعیت خوردن هدونیک بررسی می شود. بنابراین مکمل ملاتونین یا دارونما به مدت 8 هفته برای شما تجویز می گردد.

**چرا من انتخاب شده ام؟**

چون شما دارای اضافه وزن و یا چاقی هستید، شدت خوردن هدونیک در شما بالاست و شرایط ورود به مطالعه را دارید و میتوانید در صورت تمایل به همکاری در این مطالعه شرکت نمایید.

**منافع اين تحقيق چيست؟**

نتایج حاصل از این مطالعه میتواند تاثیر مکملیاری ملاتونین را بر خوردن هدونیک و کاهش وزن در شما مشخص نماید. این اطلاعات میتواند جهت انجام مطالعات آتی مورد استفاده قرار گرفته و احتمالا با توجه به آنها بتوان راهکارهایی را جهت برطرف نمودن چاقی و اضافه وزن ارائه کرد.

**آيا خطر ويا عوارض احتمالي نيز در كار خواهد بود؟** **( اگر بلی مدیریت، پیگیری و جبران عوارض به صورت رایگان خواهد بود)**

خیر. هیچ نوع عارضه ای متوجه افراد شرکت کننده در این مطالعه نخواهد بود. در صورت نیاز می توانید با شماره ای که در پایین صفحه قید شده است تماس حاصل فرمایید.

**آيا شركت من در اين مطالعه محرمانه خواهد ماند؟**

شركت شما در اين مطالعه و اطلاعات / داده هايي كه شما در اختيار من مي گذاريد، كاملا" محرمانه باقي خواهد ماند. يك شماره و يا كد شناسايي در طول مطالعه براي هر يك از شركت كنندگان اختصاص يافته و تمام داده ها ناشناخته باقي خواهند ماند. در مورد داده ها مطابق با قوانين مراقبت از داده ها در ايران كه محرمانه بودن آنها را تضمين مي كند عمل خواهد شد.

**اگر بخواهم شركت كنم چه كاري بايد انجام دهم؟**

اگر شما براي شركت در اين مطالعه موافقت كنيد، لازم است فرم رضايت آگاهانه را تكميل نموده و به محقق برگردانيد. لطفا" اين برگ حاوي اطلاعات را براي خود نگهداريد. اگر تصميم به شركت در اين تحقيق گرفتيد، هر زمانی شما مجاز هستيد از اين تحقيق كناره گيري كنيد بدون اينكه دليلي براي ما اقامه نمائيد.

**در اين بخش به طورخلاصه توضيحی درمورد نحوه مشارکت و نقش مشارکت کننده در تحقيق داده میشود:** شما به مدت 8 هفته از مکمل ملاتونین یا دارونما استفاده خواهید کرد. در ابتدا و انتهای مطالعه سنجش برخی از شاخص های خونی و اندازه گیری قد، وزنَ، BMI و ترکیب بدن برای شما انجام خواهد شد و پرسشنامه هایی در رابطه با عادات خواب و خوراک توسط شما تکمیل خواهد شد.

اگر شما سوالي داريد و يا اينكه مايل به اطلاعات بيشتري هستيد، لطفا با محقق به شماره تلفن: 09050850526 و یا E-mail : m.karamizadeh99@gmail.com تماس بگيريد. در صورت بروز مشکلی در روند شرکت شما در این طرح می توانید با کارشناس کمیته اخلاق معاونت تحقیقات و فناوری به شماره تلفن 33370119 تماس بگیرید. با تشكر از وقت شما براي قبول زحمت خواندن اين برگ حاوي اطلاعات.

**برگ دوم**

**رضايت آگاهانه**

**كد / شماره مطالعاتي:**

**عنوان تحقيق:**

لطفا" علامت گذاري كنيد:

1- من تائيد مي كنم كه برگ اطلاعات مشاركت كننده به تاريخ را براي انجام تحقيق فوق خوانده و فهميده ام و اين فرصت براي من داده شده كه سوالات مورد نظرم را بپرسم. □

2- من ميدانم كه شركت من در اين تحقيق داوطلبانه است. من همچنين مي دانم كه من هر زماني كه بخواهم مي توانم از تحقيق كنار بكشم بدون اينكه ملزم به ارائه دليل باشم. □

3- من موافقت مي كنم كه در مطالعه/ تحقيق فوق شركت نمايم. □

نام مشاركت كننده تاريخ: امضاء

نام محقق تاريخ: امضاء

رونوشت:

- مشاركت كننده
- محقق
