# Supplementary material for: The effect of melatonin supplementation on the plasma levels of 2-arachidonoylglycerol, ghrelin and hedonic eating intensity in overweight/obese females: A study protocol for a pilot randomized controlled trial
Source: PLoS One. 2025 Apr 22;20(4):e0319258. doi: 10.1371/journal.pone.0319258 (PMC12013906; doi:10.1371/journal.pone.0319258)
Supplement: S2 File — (DOCX) [file pone.0319258.s003.docx]

بسمه تعالي

##### [
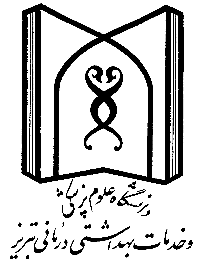
](http://www.google.com/url?sa=i&rct=j&q=&esrc=s&source=images&cd=&cad=rja&uact=8&ved=0ahUKEwi73NXWurfNAhWFlCwKHd4vCkIQjRwIBw&url=http://sama.tbzmed.ac.ir/&psig=AFQjCNHH6qa8FP5bdOG_bsc3u9KmtVISfw&ust=1466541418394654)

Tabriz University of Medical Sciences

**معاونت پژوهشی**

**Research Deputy**

**" طرح پیشنهادی تحقیق"**

**Research proposal**

**عنوان طرح:** تاثیر مکمل یاری ملاتونین بر سطوح پلاسمایی ۲-آراشیدونویل گلیسرول، گرلین و شدت خوردن لذت در زنان دارای اضافه وزن و یا چاقی

**Title:** The effect of melatonin supplementation on the plasma levels of 2-arachidonoylglycerol, ghrelin and hedonic eating intensity in overweight/obese females

**نام و نام خانوادگي مجري/ استاد راهنمای اول:** دکتر رضا مهدوی

**Conductor of the research project/ Supervisor:**

Prof. Reza Mahdavi

Prof. Bahram Pourghassem Gargari

**دانشکده: تغذیه و علوم غذایی**

Faculty of Nutrition and Food Sciences

1. **مقدمه، بيان مسئله و ضرورت اجرای طرح:**

اضافه وزن و چاقی از مشکلات بهداشت عمومی جهانی محسوب می­شوند. سازمان بهداشت جهانی افزایش تقریباً سه برابری در شیوع چاقی را در چهار دهه گذشته (بین سال‌های 1975 تا 2016) به ویژه در بین زنان ثبت کرده است (1). اگر این روند به طور مداوم ادامه یابد، تا سال 2030، 38 درصد از جمعیت بزرگسال جهان اضافه وزن خواهند داشت و 20 درصد دیگر چاق خواهند بود (2). در ایران طبق مطالعه ای که امین و همکاران در سال 2021 بر روی حدود 30 هزار بزرگسال انجام دادند، شیوع چاقی و اضافه وزن به ترتیب 23 و 37 درصد برآورد شد (3). چاقی با افزایش قابل توجه بافت چربی ناشی از عدم تعادل در دریافت غذا و مصرف انرژی مشخص می­شود (4). علاوه بر عوارض جسمی نظیر بیماری­های قلبی عروقی، بیماری­های نورولوژیک، برخی از سرطان ها، بیماری­های تنفسی، بیماری­های دستگاه گوارش، بیماری­های کلیوی، بیماری­های عضلانی اسکلتی، بیماری­های سیستم ایمنی که بسیاری از آنها می­توانند منجر به مرگ و میر شوند، چاقی منجر به عوارض روانی- اجتماعی مانند تصویر بدنی منفی، اعتماد به نفس پایین، انزوای اجتماعی، افسردگی، اضطراب و استرس نیز می­شود (5-7).

اگرچه عوامل بسیاری از جمله ژنتیک و سبک زندگی کم تحرک امروزی منجر به افزایش وزن بیش از حد می­شوند، اما تغییر عادات غذایی نقش بسزایی در افزایش خطرناک چاقی داشته است (8, 9). دریافت غذا، به ویژه در دوران مدرن که غذاهای لذیذ و پر انرژی، ارزان و همه جا در دسترس هستند، اغلب در غیاب نیاز به کالری نیز رخ می­دهد و این موضوع می­تواند برای کنترل وزن مضر باشد (10, 11). در واقع دریافت غذا و اشتها، عملکردهای پیچیده ای هستند که تا حدودی توسط دو سیستم بسیار مرتبط به هم کنترل می­شوند: سیستم هموستاتیک و سیستم هدونیک (12). در خوردن هموستاتیک، کمبود انرژی باعث فعال شدن واسطه های گرسنگی هیپوتالاموس می­شود که باعث افزایش مصرف غذا می­شود. این به نوبه خود منجر به انتشار سیگنال های سیری هیپوتالاموس می­شود که مصرف غذا را متوقف می­کند. در مقابل، در خوردن هدونیک، مسیر پیشنهادی به این صورت است که مواجهه با غذاهای لذیذ، مدارهای پاداش مزولیمبیک مغز را فعال می­کند که باعث تحریک مداوم سیگنال های گرسنگی هیپوتالاموس و مهار واسطه های سیری می­شود. در این شرایط میل به غذا خوردن حفظ می­شود و غذا بدون نیاز به انرژی و صرفا به دلیل خواص پاداش دهندگی و لذیذ بودن مصرف می­شود (13). فعال شدن مدارهای پاداش مزولیمبیک مغز در نتیجه دریافت غذاهای لذیذ در فرآیند خوردن هدونیک، به واسطه آزاد شدن دوپامین صورت می­پذیرد و دوپامین مسئول احساس خوشایند مرتبط با مصرف غذاهای لذیذ است. همچنین این نوع غذاها می توانند منجر به آزاد شدن اندوکانابینوئیدها و اوپیوئیدها شوند که این مواد نیز می توانند به افزایش آزاد شدن دوپامین کمک کنند (13-15). اندوکانابینوئیدها جزئی از سیستم اندوکانابینوئید هستند. در واقع سیستم اندوکانابینوئید از گیرنده های کانابینوئیدی شامل CB1 (Cannabinoid receptor type1) و CB2 (Cannabinoid receptor type2) و لیگاندهای اندوژن آنها از جمله اندوکانابینوئیدهای 2-آراشیدونیل گلیسرول (2-AG) و آناندامید (N-آراشیدونیل اتانول آمین) تشکیل شده است. این سیستم یک فاکتور مهم در دریافت غذا و خوردن هدونیک است. بدین صورت که گیرنده های CB1 در سیستم لیمبیک، توسط اندوکانابینوئیدها که در نتیجه مصرف غذاهای لذیذ آزاد می­شوند، فعال می شوند. فعال شدن گیرنده های CB1 در سیستم لیمبیک می تواند با تحریک آزاد شدن دوپامین، دریافت غذا و به ویژه خوردن هدونیک را افزایش دهد (16-18). اپیوئیدها نیز می توانند باعث افزایش آزاد شدن دوپامین در مدارهای پاداش مغزی شوند و در نتیجه خوردن هدونیک را افزایش دهند (13, 19). همچنین، علاوه بر نقشی که گرلین در تنظیم هموستاتیک دریافت غذا دارد، تحقیقات نشان داده اند که گرلین در پاسخ به مصرف غذاهای لذیذ در شرایط سیری به جای کاهش، افزایش می­یابد و باعث تحریک آزاد شدن دوپامین، افزایش فعالیت گیرنده های دوپامینی و تأثیر بر گیرنده های اپیوئیدی درگیر در پاداش غذا می­شود و بنابراین نقش مهمی در خوردن هدونیک دارد (20-22). در مطالعه انجام شده توسط Monteleon و همکاران، مصرف غذا برای لذت باعث افزایش سطوح گرلین و 2-آراشیدونیل گلیسرول شد و سطوح این دو همبستگی مثبتی را نشان دادند (13). خوردن هدونیک در زنان نسبت به مردان بالاتر است (23) و با توجه به نقش مهمی که در توسعه چاقی در دهه‌های اخیر دارد، در مداخلات مدیریت وزن اقداماتی که منجر به کاهش خوردن هدونیک شده است با کاهش وزن بیشتر همراه بوده است (24, 25).

علی رغم اهمیت خوردن هدونیک در مبحث اضافه وزن و چاقی، طبق بررسی های انجام شده، مداخلات زیادی جهت کاهش خوردن هدونیک طراحی نشده است. تحقیقات قبلی نشان دادند که دارویی به نام ریمونابانت به عنوان آنتاگونیست CB1 می­تواند بر خوردن هدونیک موثر باشد اما به علت اثرات منفی بر خلق و خو، پس از مدتی از بازار جمع آوری شد (26). در مطالعه Beaumont و همکاران در سال ۲۰۲۱ که بر روی افراد با وزن نرمال انجام شد، اتصال جریان 2 میلی آمپر به مدت 20 دقیقه به ناحیه PFC (prefrontal cortex) که در کنترل خوردن هدونیک نقش دارد، تأثیری بر خوردن هدونیک نداشت (27). همچنین Mason و همکاران در سال 2016 مداخله ای آموزش محور با هدف ارتقای خوردن آگاهانه طراحی نمودند که پس از 6 ماه توانست خوردن هدونیک را در افراد کاهش دهد (28). در مطالعه انجام شده توسط Montelius و همکاران در سال 2014، مکمل­یاری تیلاکوئید برگرفته از عصاره اسفناج به مدت 3 ماه توانست خوردن هدونیک را کاهش دهد (29). علاوه بر این موارد، با توجه به نتایج مطالعات حیوانی انجام شده، به نظر می رسد مکمل­یاری ملاتونین نیز بتواند خوردن هدونیک را کاهش دهد. ملاتونین هورمونی است که از غده پینه آل ترشح می­شود و تحقیقات نقش­های متعددی را برای آن نشان داده­اند. به طور مثال در تنظیم خواب و ریتم شبانه روزی نقش دارد، فعالیت آنتی اکسیدانی و ضد التهابی دارد و در کاهش وزن موثر است (32-30). نتایج مطالعه حیوانی انجام شده توسط طاهری و همکاران، کاهش در سطح سرمی گرلین را پس از مکمل­یاری ملاتونین نشان داد (33). همچنین در مطالعه حیوانی انجام شده توسط Piccinetti و همکاران، مکمل یاری ملاتونین توانست باعث کاهش بیان ژن هورمون گرلین، کاهش بیان ژن گیرنده CB1 و کاهش دریافت غذا شود (18). همانطور که قبلا اشاره شد، گرلین و اندوکانابینوئیدها در فرآیند خوردن هدونیک نقش دارند. لذا مکمل­یاری ملاتونین احتمالا می تواند با کاهش بیان ژن هورمون گرلین، کاهش سطح سرمی گرلین و کاهش بیان ژن گیرنده CB1، دریافت غذا و خوردن هدونیک را کاهش دهد. طبق بررسی های صورت گرفته، تاکنون هیچ مطالعه انتشار یافته ای به طور خاص اثر مکمل­یاری ملاتونین بر خوردن هدونیک را بررسی نکرده است.

1. **Introduction**

Overweight and obesity are considered global public health problems. The World Health Organization has recorded an almost three-fold increase in the prevalence of obesity over the past four decades (between 1975 and 2016), especially among women (1). If this trend continues, by 2030, 38% of the world’s adult population will be overweight, and another 20% will be obese (2). In Iran, according to a study conducted by Amin et al. in 2021 on about 30,000 adults, the prevalence of obesity and overweight was estimated at 23% and 37%, respectively (3). Obesity is characterized by a significant increase in adipose tissue caused by an imbalance in energy intake and energy consumption (4). The physical effects of obesity include cardiovascular diseases, neurological diseases, some cancers, respiratory diseases, gastrointestinal diseases, kidney diseases, musculoskeletal diseases, and immune system diseases, many of which can lead to death. Obesity also leads to psychosocial complications such as negative body image, low self-confidence, social isolation, depression, anxiety, and stress (5-7).

Although many factors, including genetics and today’s sedentary lifestyle, lead to excessive weight gain, changing eating habits has played a significant role in the dangerous increase in obesity (8, 9). Food intake, especially in the modern era when palatable and energy-dense foods are cheap and ubiquitous, often occurs without caloric need, which can be detrimental to weight control (10, 11). Indeed, food intake and appetite are complex functions controlled in part by two highly interrelated systems: the homeostatic system and the hedonic system (12). In homeostatic eating, the lack of energy causes the activation of hypothalamic hunger mediators, which increases food intake. This, in turn, leads to the release of satiety signals from the hypothalamus, which stops food intake. On the other hand, in hedonic eating, the proposed pathway is that exposure to palatable foods activates the brain’s mesolimbic reward circuits, which causes continuous stimulation of hypothalamic hunger signals and inhibits satiety mediators. In this condition, the desire to eat is preserved, and food is consumed without the need for energy and simply because of its rewarding and palatable properties (13).

The activation of brain mesolimbic reward circuits as a result of consuming palatable foods during hedonic eating occurs through the release of dopamine. Dopamine is responsible for the pleasant feeling associated with consuming palatable foods. These types of foods can also lead to the release of endocannabinoids and opioids, which further help increase the release of dopamine (13-15). Endocannabinoids are part of the endocannabinoid system. The endocannabinoid system consists of cannabinoid receptors, including Cannabinoid Receptor Type 1 (CB1) and Cannabinoid Receptor Type 2 (CB2), and their endogenous ligands, such as endocannabinoids 2-arachidonoylglycerol (2-AG) and anandamide (N-arachidonylethanolamine). This system is an important factor in food intake and hedonic eating. CB1 receptors in the limbic system are activated by endocannabinoids released as a result of eating palatable foods. Activation of CB1 receptors in the limbic system can increase food intake, especially hedonic eating, by stimulating the release of dopamine (16-18). Opioids can also increase the release of dopamine in brain reward circuits, thereby enhancing hedonic eating (13, 19). Additionally, in addition to the role of ghrelin in the homeostatic regulation of food intake, research has shown that ghrelin increases in response to the consumption of palatable foods in satiety conditions instead of decreasing. It stimulates the release of dopamine, increases the activity of dopamine receptors, and affects opioid receptors involved in food reward, thus playing an important role in hedonic eating (20-22). In the study conducted by Monteleone et al., the consumption of food for pleasure increased the levels of ghrelin and 2-arachidonoylglycerol, and the levels of these two showed a positive correlation (13).

Hedonic eating is higher in women than in men (23) and considering its significant role in the development of obesity in recent decades, weight management interventions that reduce hedonic eating have been associated with greater weight loss (24, 25). Despite the importance of hedonic eating in the context of overweight and obesity, many interventions have not been designed to address it. Previous research showed that a drug called Rimonabant, a CB1 antagonist, can be effective in reducing hedonic eating, but it was withdrawn from the market due to negative effects on mood (26). In a study by Beaumont et al., transcranial direct current stimulation targeting the dorsolateral prefrontal cortex—known for its role in controlling hedonic eating—did not impact hedonic appetite when administered at 2 mA for 20 minutes (27). Additionally, in 2016, Mason et al. designed an education-based intervention aimed at promoting mindful eating, which reduced hedonic eating in subjects after 6 months (28). In a study conducted by Montelius et al. in 2014, thylakoid supplementation derived from spinach extract for 3 months was able to reduce hedonic eating (29). In addition, according to the results of animal studies, it seems that melatonin supplementation can also reduce hedonic eating. Melatonin is a hormone secreted by the pineal gland, and research has shown several roles for it. For example, it plays a role in regulating sleep and circadian rhythm, has antioxidant and anti-inflammatory activity, and is effective in weight loss (30-32). The results of an animal study conducted by Taheri et al. showed a decrease in the serum level of ghrelin after melatonin supplementation (33). Additionally, in an animal study conducted by Piccinetti et al., melatonin supplementation decreased the expression of the ghrelin hormone gene, reduced the expression of the CB1 receptor gene, and decreased food intake (18). As mentioned earlier, ghrelin and endocannabinoids are involved in the hedonic eating. Therefore, melatonin supplementation can probably reduce food intake and hedonic eating by reducing the expression of the ghrelin hormone gene, reducing the serum level of ghrelin, and reducing the expression of the CB1 receptor gene. According to reviews, no published study has specifically investigated the effect of melatonin supplementation on hedonic eating.

**2- سؤالات یا فرضيات طرح (HYPOTHESES/RESEARCH QUESTIONS) :‌**

1) مکمل یاری ملاتونین بر سطوح سرمی 2-آراشیدونیل گلیسرول و گرلین در زنان اضافه وزن/ چاق با امتیاز بالای خوردن هدونیک مؤثر است.

2) مکمل یاری ملاتونین بر امتیاز خوردن هدونیک در زنان اضافه وزن/ چاق با امتیاز بالای خوردن هدونیک مؤثر است.

3) مکمل یاری ملاتونین بر وضعیت تغذیه ای (دریافت انرژی کل، دریافت انرژی از ماکرونوترینت ها و وزن) زنان اضافه وزن/ چاق با امتیاز بالای خوردن هدونیک مؤثر است.

Hypothesis 1: Melatonin supplementation is effective on the serum levels of 2-arachidonylglycerol and ghrelin in overweight/obese women with high hedonic eating intensity.

Hypothesis 2: Melatonin supplementation is effective on hedonic eating intensity in overweight/obese women with high hedonic eating intensity.

Hypothesis 3: Melatonin supplementation is effective on the nutritional status (total energy intake, energy intake from macronutrients, and weight) of overweight/obese women with high hedonic eating intensity.

**3- اهداف طرح (RESEARCH OBJECTIVES)**

**الف) هدف كلي طرح (AIM)**:

تعیین اثر مکمل یاری ملاتونین بر سطوح خونی2-آراشیدونیل گلیسرول، گرلین و شدت خوردن هدونیک در زنان اضافه وزن/ چاق

Determining the effect of melatonin supplementation on plasma levels of 2-arachidonylglycerol, ghrelin, and hedonic eating intensity in overweight/obese females.

**ب) اهداف اختصاصي طرح (SPECIFIC OBJECTIVE):**

1) تعیین و مقایسه اثر مکمل یاری ملاتونین و دارونما بر سطوح سرمی 2-آراشیدونیل گلیسرول و گرلین در زنان اضافه وزن/ چاق با امتیاز بالای خوردن هدونیک

2) تعیین و مقایسه اثر مکمل یاری ملاتونین و دارونما بر امتیاز خوردن هدونیک در زنان اضافه وزن/ چاق با امتیاز بالای خوردن هدونیک

3) تعیین و مقایسه اثر مکمل یاری ملاتونین و دارونما بر وضعیت تغذیه ای (دریافت انرژی کل، دریافت انرژی از ماکرونوترینت ها و وزن) در زنان اضافه وزن/ چاق با امتیاز بالای خوردن هدونیک

1) Determining and comparing the effects of melatonin supplementation and placebo on plasma levels of 2-arachidonylglycerol and ghrelin in overweight/obese women with high hedonic eating intensity.

2) Determining and comparing the effects of melatonin supplementation and placebo on hedonic eating intensity in overweight/obese women with high hedonic eating intensity.

3) Determining and comparing the effects of melatonin supplementation and placebo on nutritional status (total energy intake, energy intake from macronutrients, and weight) in overweight/obese women with high hedonic eating intensity.

**4- روش اجرا** **(Procedure):**

مطالعه حاضر فاز دوم پایان نامه مقطع دکتری می باشد. مرحله اول این پروژه مقطعی است و تقریباً 400 زن دارای اضافه وزن یا چاقی (BMI: 25-39.9 کیلوگرم بر متر مربع) از طریق تبلیغات در مکان های عمومی، رسانه های اجتماعی، مراکز تناسب اندام و ... استخدام خواهند شد. شدت خوردن هدونیک بالا با استفاده از مقیاس قدرت غذا (PFS) که قبلاً روایی و پایایی آن در جمعیت ایرانی تایید شده است، ارزیابی خواهد شد. داوطلبانی که از نمره آستانه فراتر می روند (یعنی نمره کل PFS > 2.5)، به مرحله بعدی تحقیق که شامل مداخله است، وارد می شوند. همه شرکت‌کنندگان واجد شرایط، اطلاعات جامعی در مورد پروسه تحقیق توسط محقق اصلی دریافت خواهند کرد و رضایت آنان با امضای یک فرم رضایت آگاهانه کسب خواهد شد.

مطالعه حاضر به صورت کارآزمایی بالینی تصادفی کنترل دار دوسوکور خواهد بود و زنان بزرگسالی که معیارهای ورود به مطالعه زیر را داشته باشند، استخدام خواهند شد: داوطلبان ظاهرا سالم، باسواد، 19 تا 49 سال. BMI = 25-39.9 کیلوگرم بر متر مربع؛ نشان دادن شدت خوردن هدونیک زیاد (همانطور که توسط مقیاس قدرت غذا (PFS) با نمره کل PFS > 2.5 تعیین می‌شود). و ابراز تمایل به استفاده از داروها یا مکمل ها برای کاهش وزن به جای رژیم گرفتن.

شرکت کنندگان بر اساس معیارهای زیر حذف خواهند شد: یائسه بودن، باردار بودن یا شیردهی. داشتن قاعدگی نامنظم؛ کاهش وزن اخیر یا شرکت در برنامه های کاهش وزن؛ وضعیت فعلی سیگار کشیدن؛ استفاده اخیر از مکمل های موثر بر اشتها یا وزن (در 3 ماه گذشته)؛ سوء مصرف مواد؛ مصرف الکل؛ برخی شرایط پزشکی مانند سرطان، بیماری قلبی و دیابت؛ کم کاری تیروئید تازه تشخیص داده شده یا کنترل نشده؛ استفاده از داروهای مؤثر بر اشتها (مانند داروهای ضد افسردگی، استروئیدها و داروهای ضد بارداری خوراکی)؛ کار در شیفت شب؛ بی خوابی شدید [همانطور که توسط شاخص شدت بی خوابی (ISI) تعیین می شود]؛ استفاده از داروهایی که با ملاتونین تداخل دارند (مانند داروهای ضد تشنج، داروهای ضد انعقاد، آرام بخش ها). عدم تحمل مکمل ملاتونین در طول مطالعه؛ یا استفاده از کمتر از 90 درصد از مکمل های ملاتونین یا دارونما ارائه شده است.

در شروع مطالعه، پس از کسب رضایت نامه کتبی آگاهانه از شرکت کنندگان، پرسشنامه اطلاعات دموگرافیک، تاریخچه پزشکی و دارویی به صورت خود گزارش دهی تکمیل خواهند شد.

در حال حاضر، هیچ مطالعه انسانی استفاده از مکمل ملاتونین را در خوردن هدونیک بررسی نکرده است. مطالعه پیشنهادی یک پروژه آزمایشی است. هدف اصلی آن ایجاد داده هایی برای محاسبات حجم نمونه برای مداخلات بزرگتر است. مطالعات مختلف حجم نمونه کل حداقل 24 شرکت کننده را برای یک کارآزمایی دو بازویی توصیه می کنند. در این کارآزمایی بالینی، قصد داریم 40 شرکت‌کننده را ثبت‌نام کنیم که با محاسبه 15 درصد ریزش، به 46 نفر (23 نفر در گروه مکمل ملاتونین و 23 نفر در گروه دارونما) افزایش می‌یابد.

افراد در دو گروه مکمل و دارونما، یک قرص 5 میلی گرمی ملاتونین یا دارونما (شامل ترکیبی از سلیکون دی اکساید، سلولوز و نشاسته) را یک ساعت قبل از خواب شبانه، به مدت 8 هفته مصرف خواهند کرد. قبل از آغاز مداخله و پس از 8 هفته از شروع مصرف مکمل یا دارونما، نمونه های خون در حالت ناشتا جمع آوری خواهند شد و سطوح سرمی 2-آراشیدونیل گلیسرول و گرلین و به روش الایزا اندازه گیری خواهند شد. همچنین برای سنجش میزان تاثیر مداخله بر وضعیت تغذیه ای، قبل از آغاز مداخله، پس از 4 هفته و پس از 8 هفته از شروع مصرف مکمل یا دارونما، وزن با استفاده از ترازوی Seca توسط محقق اندازه گیری خواهد شد و پرسشنامه یادآمد خوراک 3 روزه تکمیل خواهند شد. برای سنجش میزان تاثیر مداخله بر خوردن هدونیک، پرسشنامه قدرت غذا نیز در پایان مداخله مجددا تکمیل خواهد شد. علاوه بر این برای کنترل عوامل مداخله‌گر، فعالیت بدنی با استفاده از پرسشنامه بین‌المللی فعالیت بدنی (IPAQ) در ابتدا و انتهای مطالعه ارزیابی خواهد شد.

از نرم افزار SPSS-26 جهت تجزیه و تحلیل داده ها استفاده خواهد شد و مقادیر P-value<0.05 از نظر آماری معنادار در نظر گرفته خواهند شد. نرمال بودن توزیع داده های مورد مطالعه با استفاده از آزمون کولموگروف-اسمیرنوف بررسی خواهد شد و و تبدیل log برای نرمال سازی متغیرهایی که از توزیع نرمال پیروی نمی کنند اعمال می شود. متغیرهای کمی به صورت میانگین و انحراف معیار گزارش خواهند شد. متغیرهای کیفی به صورت فراوانی و درصد ارائه خواهند گردید. آزمون های آماری مورد استفاده در مطالعه در صورت نرمال بودن توزیع داده ها شامل آزمون­های t مستقل، t زوجی، تحلیل کوواریانس و تحلیل واریانس با اندازه گیری های مکرر خواهد بود.

The present study is the second phase of a Ph.D. dissertation. The first phase of the project is cross-sectional, and approximately 400 overweight or obese females (BMI: 25–39.9 kg/m²) will be recruited through advertisements in public places, social media, fitness centers, etc. The high hedonic eating intensity will be assessed using the Power of Food Scale (PFS), which has previously been validated in the Persian population. Volunteers who exceed the threshold score, meaning a total PFS score > 2.5, will proceed to the next phase of the research, which involves intervention. All eligible participants will receive comprehensive information regarding the research procedures by the principal investigator, and consent will be obtained by signing an informed consent document.

The present interventional study is a two-arm, parallel-group, randomized, double-blind, and placebo-controlled superiority clinical trial. For the current study, adult women meeting the following inclusion criteria will be recruited: apparently healthy volunteers, literate, aged 19–49 years; BMI = 25-39.9 kg/m²; exhibiting high hedonic eating intensity (as determined by the Power of Food Scale (PFS) with total PFS score > 2.5); and expressing a desire to use drugs or supplements for weight loss instead of dieting.

Participants will be excluded based on the following criteria: being postmenopausal, pregnant, or lactating; having irregular menstruation; recent weight loss or participation in weight loss programs; current smoking status; recent use of supplements affecting appetite or weight (within the last 3 months); substance abuse; alcohol consumption; certain medical conditions such as cancer, heart disease, and diabetes; newly diagnosed or uncontrolled hypothyroidism; use of appetite-affecting drugs (such as antidepressants, steroids, and oral contraceptives); working night shifts; severe insomnia [as determined by the Insomnia Severity Index (ISI)]; use of drugs that interfere with melatonin (such as anticonvulsant drugs, anticoagulant drugs, sedatives); intolerance to melatonin supplementation during the study; or using less than 90% of the provided melatonin supplements or placebo.

At the beginning of the study, after obtaining written informed consent from the participants, questionnaires on demographic information and medical and pharmaceutical history will be completed through self-reporting.

Currently, no human studies have investigated the use of melatonin supplementation on hedonic eating. The proposed study is a pilot project; its main aim is to establish data for sample size calculations for a larger trial. Various studies recommend a total sample size of at least 24 participants for a two-arm trial. In this clinical trial, we plan to enroll 40 participants, which, with a 15% attrition rate, will increase to 46 subjects (23 in the melatonin group and 23 in the placebo group).

Participants in both the supplement and placebo groups will take a 5 mg melatonin tablet or a placebo (a combination of silicon dioxide, cellulose, and starch) one hour before going to bed at night for 8 weeks. Before the start of the intervention and after 8 weeks of taking the supplement or placebo, fasting blood samples will be collected, and plasma levels of 2-arachidonylglycerol and ghrelin will be measured using ELISA kits. Additionally, to measure the impact of the intervention on nutritional status, weight will be measured by the researcher using a Seca scale before the intervention, after 4 weeks, and after 8 weeks from the start of taking the supplement or placebo, and three 24-hour dietary recalls will be completed. To measure the impact of the intervention on hedonic eating, the Power of Food Scale will be completed again at the end of the intervention. Moreover, as a means of controlling for confounding factors, the validated short form of the Persian version of the International Physical Activity Questionnaire (IPAQ) will be used to evaluate physical activity at both the study’s outset and its completion.

Data will be analyzed statistically using SPSS 26.0 software, considering P values below 0.05 as indicative of statistical significance. The normality of data distribution will be checked using the Kolmogorov-Smirnov test, and log transformation will be applied to normalize variables that do not follow a normal distribution. Mean (standard deviation) and frequency (percentage) will be used to represent quantitative and qualitative variables, respectively. If the data distribution is normal, the statistical tests used in the study will include independent sample t-tests, paired-sample t-tests, repeated measures ANOVA, and analysis of covariance (ANCOVA).

**5– توضیحات تکمیلی اهداف اختصاصی (Additional explanations of specific objectives):**

**هدف اختصاصي شماره 1: تعیین و مقایسه اثر مکمل یاری ملاتونین و دارونما بر سطوح سرمی 2-آراشیدونیل گلیسرول و گرلین در زنان اضافه وزن/چاق با امتیاز بالای خوردن هدونیک**

**1) Determining and comparing the effects of melatonin supplementation and placebo on plasma levels of 2-arachidonylglycerol and ghrelin in overweight/obese women with high hedonic eating intensity.**

الف) خلاصه روش اجرا (Procedure Summary):

جهت تعیین و مقایسه اثر مکمل یاری ملاتونین و دارونما بر بر سطوح سرمی گرلین و 2-آراشیدونیل گلیسرول در زنان با امتیاز بالای خوردن هدونیک در 46 زن با امتیاز بالای خوردن هدونیک (23 نفر در گروه مکمل ملاتونین و 23 نفر در گروه دارونما)، قبل از شروع مطالعه و در انتهای مطالعه، از هر شرکت کننده صبح پس از یک ناشتایی 10-12 ساعته، 5 سی سی نمونه خون وریدی از بازوی چپ گرفته شده، با استفاده از سانتریفیوژ پلاسما جدا گردیده و تا زمان اندازه گیری سطوح پلاسمایی گرلین و 2-آراشیدونیل گلیسرول در دمای 80- درجه سانتی گراد نگه داری خواهد شد. سپس سطوح پلاسمایی گرلین و 2-آراشیدونیل گلیسرول با استفاده از روش الایزا اندازه گیری خواهد شد.

To determine and compare the effect of melatonin supplementation and placebo on the plasma levels of 2-arachidonylglycerol and ghrelin in 46 women with a high hedonic eating intensity (23 in the melatonin supplement group and 23 in the placebo group), 5 cc of venous blood samples will be taken from the left arm of each participant in the morning after a 10-12 hour fast, both before the start of the study and at its end. The plasma will be separated using a centrifuge and stored at -80 degrees Celsius. Then, the plasma levels of ghrelin and 2-arachidonyl glycerol will be measured using ELISA kits.

ب) توضیح کامل روش تجزيه و تحليل آماري داده ها (Statistical Analysis Method):

این هدف با استفاده از نرم افزار SPSS تجزیه و تحلیل خواهد شد. جهت مقایسه سطوح پلاسمایی گرلین و 2-آراشیدونیل گلیسرول بین گروه مکمل ملاتونین و گروه دارونما، قبل از شروع مداخله از آزمون تی مستقل استفاده خواهد شد. جهت مقایسه سطوح پلاسمایی گرلین و 2-آراشیدونیل گلیسرول بین گروه مکمل ملاتونین و گروه دارونما، قبل از شروع مداخله و در انتهای مداخله از آزمون تی زوجی استفاده خواهد شد و جهت مقایسه سطوح پلاسمایی گرلین و 2-آراشیدونیل گلیسرول بین گروه مکمل ملاتونین و گروه دارونما، در انتهای مداخله از آزمون تحلیل کوواریانس استفاده خواهد شد.

This specific objective will be analyzed using SPSS software. To compare the plasma levels of 2-arachidonylglycerol and ghrelin between the melatonin group and the placebo group, an independent sample t-test will be used before the intervention. To compare the plasma levels of 2-arachidonylglycerol and ghrelin within the melatonin group and the placebo group, before the start of the intervention and at the end of the intervention, a paired sample t-test will be used. To compare the plasma levels of 2-arachidonylglycerol and ghrelin between the melatonin group and the placebo group at the end of the intervention, ANCOVA will be used.

**هدف اختصاصي شماره 2: تعیین و مقایسه اثر مکمل یاری ملاتونین و دارونما بر امتیاز خوردن هدونیک در زنان با امتیاز بالای خوردن هدونیک**

**2) Determining and comparing the effects of melatonin supplementation and placebo on hedonic eating intensity in overweight/obese women with high hedonic eating intensity.**

الف) خلاصه روش اجرا (Procedure Summary):

جهت تعیین و مقایسه اثر مکمل یاری ملاتونین و دارونما بر امتیاز خوردن هدونیک در زنان با امتیاز بالای خوردن هدونیک در 46 زن با امتیاز بالای خوردن هدونیک (23 نفر در گروه مکمل ملاتونین و 23 نفر در گروه دارونما)، در فاز اول مطالعه و در انتهای مطالعه، پرسشنامه قدرت غذا تکمیل خواهد شد.

To determine and compare the effect of melatonin supplementation and placebo on hedonic eating intensity in 46 women with high hedonic eating intensity (23 subjects in the melatonin group and 23 subjects in the placebo group), the Power of Food Scale will be completed in the first phase of the project and at the end of the study.

ب) توضیح کامل روش تجزيه و تحليل آماري داده ها (Statistical Analysis Method):

این هدف با استفاده از نرم افزار SPSS تجزیه و تحلیل خواهد شد. جهت مقایسه امتیاز خوردن هدونیک بین گروه مکمل ملاتونین و گروه دارونما، قبل از شروع مداخله از آزمون تی مستقل استفاده خواهد شد. جهت مقایسه امتیاز خوردن هدونیک بین گروه مکمل ملاتونین و گروه دارونما، قبل از شروع مداخله و در انتهای مداخله از آزمون تی زوجی استفاده خواهد شد و جهت مقایسه امتیاز خوردن هدونیک بین گروه مکمل ملاتونین و گروه دارونما، در انتهای مداخله از آزمون تحلیل کوواریانس استفاده خواهد شد.

This specific objective will be analyzed using SPSS software. To compare the hedonic eating scores between the melatonin group and the placebo group, an independent sample t-test will be used before the intervention. To compare the hedonic eating scores within the melatonin group and the placebo group, before the start of the intervention and at the end of the intervention, a paired sample t-test will be used. To compare the hedonic eating scores between the melatonin group and the placebo group at the end of the intervention, ANCOVA will be used.

**هدف اختصاصي شماره 3: تعیین و مقایسه اثر مکمل یاری ملاتونین و دارونما بر وضعیت تغذیه ای (دریافت انرژی کل، دریافت انرژی از ماکرونوترینت ها و وزن) در زنان با امتیاز بالای خوردن هدونیک**

**3) Determining and comparing the effects of melatonin supplementation and placebo on nutritional status (total energy intake, energy intake from macronutrients, and weight) in overweight/obese women with high hedonic eating intensity.**

الف) خلاصه روش اجرا (Procedure Summary):

جهت تعیین و مقایسه اثر مکمل یاری ملاتونین و دارونما بر وضعیت تغذیه ای (دریافت انرژی کل، دریافت انرژی از ماکرونوترینت ها و وزن) در زنان با امتیاز بالای خوردن هدونیک در 46 زن با امتیاز بالای خوردن هدونیک (23 نفر در گروه مکمل ملاتونین و 23 نفر در گروه دارونما)، قبل از شروع مطالعه، 4 هفته پس از آغاز مطالعه و در انتهای مطالعه، پرسشنامه یادآمد خوراک 3 روزه تکمیل خواهد شد و سنجش وزن توسط ترازو انجام خواهد شد.

To determine and compare the effect of melatonin supplementation and placebo on nutritional status (total energy intake, energy intake from macronutrients, and weight) in 46 women with high hedonic eating intensity (23 in the melatonin group and 23 in the placebo group), three 24-hour dietary recalls will be completed, and weight will be measured using a Seca scale before the start of the study, 4 weeks after the start of the study, and at the end of the study.

ب) توضیح کامل روش تجزيه و تحليل آماري داده ها (Statistical Analysis Method):

این هدف با استفاده از نرم افزار SPSS تجزیه و تحلیل خواهد شد. جهت مقایسه دریافت انرژی، دریافت انرژی از ماکرونوترینت ها و وزن بین گروه مکمل ملاتونین و گروه دارونما، قبل از شروع مداخله از آزمون تی مستقل استفاده خواهد شد. جهت مقایسه دریافت انرژی، دریافت انرژی از ماکرونوترینت ها و وزن بین گروه مکمل ملاتونین و گروه دارونما، قبل از شروع مداخله، 4 هفته پس از آغاز مداخله و در انتهای مداخله از آزمون تحلیل واریانس با اندازه گیری های مکرر استفاده خواهد شد و جهت مقایسه دریافت انرژی، دریافت انرژی از ماکرونوترینت ها و وزن بین گروه مکمل ملاتونین و گروه دارونما، در انتهای مداخله از آزمون تحلیل کوواریانس استفاده خواهد شد.

This specific objective will be analyzed using SPSS software. An independent sample t-test will be used before the intervention to compare the nutritional status between the melatonin group and the placebo group. A repeated measures ANOVA will be used to compare the nutritional status within the melatonin group and the placebo group before the start of the intervention, 4 weeks after the beginning of the intervention, and at the end of the intervention. To compare the nutritional status between the melatonin group and the placebo group at the end of the intervention, ANCOVA will be used.

**6**- ملاحظات اخلاقي **(Ethical Considerations)**

افراد شرکت کننده در ابتدای مطالعه فرم رضایت نامه آگاهانه را تکمیل خواهند کرد و در هر مرحله از طرح حق خروج از مطالعه را دارند.

The participants will complete the informed consent form at the beginning of the study and they have the right to withdraw from the study at any stage of the project.

**7- منابع (References):**

1. Dias FSB, de Moura Magalhães Lima Y, Martins FA, da Silva-Nunes M, de Andrade AM, Ramalho AA. Time Trend of Overweight and Obesity in Adults from Rio Branco, Acre, Western Brazilian Amazon (2006-2020). Nutrients. 2022;14(4).

2. Tekalegn Y, Solomon D, Sahiledengle B, Assefa T, Negash W, Tahir A, et al. Prevalence of central obesity and its associated risk factors among adults in Southeast Ethiopia: A community-based cross-sectional study. 2022;17(8):e0265107.

3. Amin R, Kolahi A-A, Sohrabi M-RJOf. Disparities in obesity prevalence in Iranian adults: cross-sectional study using data from the 2016 STEPS survey. 2021;14(3):298-305.

4. Pivonello C, Negri M, Patalano R, Amatrudo F, Montò T, Liccardi A, et al. The role of melatonin in the molecular mechanisms underlying metaflammation and infections in obesity: A narrative review. Obesity reviews : an official journal of the International Association for the Study of Obesity. 2022;23(3):e13390.

5. Wharton S, Lau DC, Vallis M, Sharma AM, Biertho L, Campbell-Scherer D, et al. Obesity in adults: a clinical practice guideline. 2020;192(31):E875-E91.

6. Kinlen D, Cody D, O'Shea D. Complications of obesity. QJM : monthly journal of the Association of Physicians. 2018;111(7):437-43.

7. Wu YK, Berry DC. Impact of weight stigma on physiological and psychological health outcomes for overweight and obese adults: A systematic review. Journal of advanced nursing. 2018;74(5):1030-42.

8. Wiss DA, Criscitelli K, Gold M, Avena N. Preclinical evidence for the addiction potential of highly palatable foods: Current developments related to maternal influence. Appetite. 2017;115:19-27.

9. Stoś K, Rychlik E, Woźniak A, Ołtarzewski M, Jankowski M, Gujski M, et al. Prevalence and Sociodemographic Factors Associated with Overweight and Obesity among Adults in Poland: A 2019/2020 Nationwide Cross-Sectional Survey. International journal of environmental research and public health. 2022;19(3).

10. Espel-Huynh HM, Muratore AF, Lowe MR. A narrative review of the construct of hedonic hunger and its measurement by the Power of Food Scale. Obesity science & practice. 2018;4(3):238-49.

11. Unick JL, Dunsiger SI, Leblond T, Hahn K, Thomas JG, Abrantes AM, et al. Randomized Trial Examining the Effect of a 12-wk Exercise Program on Hedonic Eating. Medicine and science in sports and exercise. 2021;53(8):1638-47.

12. Berg Schmidt J, Johanneson Bertolt C, Sjödin A, Ackermann F, Vibeke Schmedes A, Lynge Thomsen H, et al. Does stress affect food preferences? - a randomized controlled trial investigating the effect of examination stress on measures of food preferences and obesogenic behavior. Stress (Amsterdam, Netherlands). 2018;21(6):556-63.

13. Monteleone P, Piscitelli F, Scognamiglio P, Monteleone AM, Canestrelli B, Di Marzo V, et al. Hedonic eating is associated with increased peripheral levels of ghrelin and the endocannabinoid 2-arachidonoyl-glycerol in healthy humans: a pilot study. The Journal of clinical endocrinology and metabolism. 2012;97(6):E917-24.

14. Nicoletti CF, Delfino HBP, Ferreira FC, Pinhel MAdS, Nonino CBJRiE, Disorders M. Role of eating disorders-related polymorphisms in obesity pathophysiology. 2019;20(1):115-25.

15. Koob G, Everitt B, Robbins T. Reward, Motivation, and Addiction. 2013. p. 871-98.

16. Aguilera Vasquez N, Nielsen DE. The Endocannabinoid System and Eating Behaviours: a Review of the Current State of the Evidence. Current Nutrition Reports. 2022;11(4):665-74.

17. Kaul M, Zee PC, Sahni AS. Effects of Cannabinoids on Sleep and their Therapeutic Potential for Sleep Disorders. Neurotherapeutics : the journal of the American Society for Experimental NeuroTherapeutics. 2021;18(1):217-27.

18. Piccinetti CC, Migliarini B, Olivotto I, Coletti G, Amici A, Carnevali O. Appetite regulation: the central role of melatonin in Danio rerio. Hormones and behavior. 2010;58(5):780-5.

19. Spagnolo PA, Kimes A, Schwandt ML, Shokri-Kojori E, Thada S, Phillips KA, et al. Striatal Dopamine Release in Response to Morphine: A [(11)C]Raclopride Positron Emission Tomography Study in Healthy Men. Biological psychiatry. 2019;86(5):356-64.

20. Buss J, Havel PJ, Epel E, Lin J, Blackburn E, Daubenmier J. Associations of ghrelin with eating behaviors, stress, metabolic factors, and telomere length among overweight and obese women: preliminary evidence of attenuated ghrelin effects in obesity? Appetite. 2014;76:84-94.

21. Edvardsson CE, Vestlund J, Jerlhag E. A ghrelin receptor antagonist reduces the ability of ghrelin, alcohol or amphetamine to induce a dopamine release in the ventral tegmental area and in nucleus accumbens shell in rats. European journal of pharmacology. 2021;899:174039.

22. Murray S, Tulloch A, Gold MS, Avena NM. Hormonal and neural mechanisms of food reward, eating behaviour and obesity. Nature reviews Endocrinology. 2014;10(9):540-52.

23. Aliasghari F, Asghari Jafarabadi M, Lotfi Yaghin N, Mahdavi R. Psychometric properties of Power of Food Scale in Iranian adult population: gender-related differences in hedonic hunger. Eating and weight disorders : EWD. 2020;25(1):185-93.

24. Shriver LH, Dollar JM, Lawless M, Calkins SD, Keane SP, Shanahan L, et al. Longitudinal Associations between Emotion Regulation and Adiposity in Late Adolescence: Indirect Effects through Eating Behaviors. Nutrients. 2019;11(3).

25. Schulte EM, Tuerk PW, Wadden TA, Garvey WT, Weiss D, Hermayer KL, et al. Changes in weight control behaviors and hedonic hunger in a commercial weight management program adapted for individuals with type 2 diabetes. International journal of obesity (2005). 2020;44(5):990-8.

.26 de Ceglia M, Decara J, Gaetani S, Rodríguez de Fonseca F. Obesity as a Condition Determined by Food Addiction: Should Brain Endocannabinoid System Alterations Be the Cause and Its Modulation the Solution? Pharmaceuticals (Basel, Switzerland). 2021;14(10).

.27 Beaumont JD, Davis D, Dalton M, Nowicky A, Russell M, Barwood MJ. The effect of transcranial direct current stimulation (tDCS) on food craving, reward and appetite in a healthy population. Appetite. 2021;157:105004.

.28 Mason AE, Epel ES, Aschbacher K, Lustig RH, Acree M, Kristeller J, et al. Reduced reward-driven eating accounts for the impact of a mindfulness-based diet and exercise intervention on weight loss: Data from the SHINE randomized controlled trial. Appetite. 2016;100:86-93.

.29 Montelius C, Erlandsson D, Vitija E, Stenblom EL, Egecioglu E, Erlanson-Albertsson C. Body weight loss, reduced urge for palatable food and increased release of GLP-1 through daily supplementation with green-plant membranes for three months in overweight women. Appetite. 2014;81:295-304.

.30 Zisapel N. New perspectives on the role of melatonin in human sleep, circadian rhythms and their regulation. British journal of pharmacology. 2018;175(16):3190-9.

.31 Delpino FM, Figueiredo LM. Melatonin supplementation and anthropometric indicators of obesity: A systematic review and meta-analysis. Nutrition (Burbank, Los Angeles County, Calif). 2021;91-92:111399.

.32 Genario R, Cipolla-Neto J, Bueno AA, Santos HO. Melatonin supplementation in the management of obesity and obesity-associated disorders: A review of physiological mechanisms and clinical applications. Pharmacological research. 2021;163:105254.

.33 Taheri P, Mogheiseh A, Shojaee Tabrizi A, Nazifi S, Salavati S, Koohi F. Changes in thyroid hormones, leptin, ghrelin and, galanin following oral melatonin administration in intact and castrated dogs: a preliminary study. BMC veterinary research. 2019;15(1):145.

- راهنماي تكميل طرح پیشنهادی تحقیق را بدقت مطالعه، ضمن موافقت با آن، صحت مطالب مندرج در آن را تأئيد مي نمايم.

**I have carefully read the guide for completing the proposed research plan. While agreeing with it, I confirm the accuracy of the contents contained therein.**

Prof. Reza Mahdavi

Prof. Bahram Pourghassem Gargari
