# Supplementary material for: The effect of melatonin supplementation on the plasma levels of 2-arachidonoylglycerol, ghrelin and hedonic eating intensity in overweight/obese females: A study protocol for a pilot randomized controlled trial
Source: PLoS One. 2025 Apr 22;20(4):e0319258. doi: 10.1371/journal.pone.0319258 (PMC12013906; doi:10.1371/journal.pone.0319258)
Supplement: Attachment — Submitted filename: Response to Reviewers.docx [file pone.0319258.s005.docx]

***Dear Dr. Marcello Iriti,***

Thank you very much for your comments on our manuscript entitled as: ***The effect of melatonin supplementation on the plasma levels of 2-arachidonoylglycerol, ghrelin and hedonic eating intensity in overweight/obese females: a study protocol for a pilot randomized controlled trial***; Manuscript ID: PONE-D-24-36320. We really appreciate your time and consideration. The following are our point-by-point responses to the comments. We have highlighted all changes in the new format of the manuscript.

**REVIEWER REPORT(S):**

**Referee: 1**

**Comments to the Author**

-) Line 101. “46 subjects (23 in each group)”. The determined sample size seems quite arbitrary. What effect size can be detected by this sample size?

As noted in p.6, lines 121-127 currently, no human studies have investigated the use of melatonin supplementation on hedonic eating. The proposed study is a pilot project; its main aim is to establish data for sample size calculations for a larger trial. Various studies recommend a total sample size of at least 24 participants for a two-arm trial. Considering the sample size of previous studies on melatonin supplementation in overweight and obese Iranian women (Mohammadi et al, https://doi.org/10.1155/2021/3502325, and Mesri Alamdari et al, http://dx.doi.org/ 10.1055/s-0034-1384587) and to increase the accuracy and power of the study, in this clinical trial, we plan to enroll 40 participants, which, with a 15% attrition rate, will increase to 46 subjects (23 in each group).

-) Line 114. “apparently healthy volunteers”. This sounds very subjective. Need to clarify how to define apparently healthy.

Apparently healthy volunteers are individuals who have not been diagnosed with any medical conditions and are not taking any medications (p.7, lines: 140-141).

-) Line 148. “if any adverse events occur, unblinding is allowed”. Please clarify whether these participants will be included in the analysis.

If dropout rates reach 15% or higher due to adverse effects, we will conduct an intention-to-treat (ITT) analysis (p.9, lines: 175-176).

-) Line 233.“log transformation will be applied to normalize variable”. As you do not know the distribution of variables yet, how do you know log-transformation will be appropriate?

It was changed to: The normality of data distribution will be checked using the Kolmogorov-Smirnov test, and an appropriate method will be applied to normalize variables that do not follow a normal distribution (p.13, lines: 272-274).

**Referee: 2**

***Comments to the Author***

-) Suggestion adding a line or two to describe the PFS briefly In the introduction section

It was added to manuscript (p.5, lines: 92-94).

-) Could you clarify how the number of 46 participants was determined? It would be helpful to provide more details on how the sample size was calculated, and whether a power analysis was conducted. Also, please explain how the 15% attrition rate was estimated.

As noted in p.6, lines 121-127 currently, no human studies have investigated the use of melatonin supplementation on hedonic eating. The proposed study is a pilot project; its main aim is to establish data for sample size calculations for a larger trial. Various studies recommend a total sample size of at least 24 participants for a two-arm trial. Considering the sample size of previous studies on melatonin supplementation in overweight and obese Iranian women (Mohammadi et al, https://doi.org/10.1155/2021/3502325, and Mesri Alamdari et al, http://dx.doi.org/ 10.1055/s-0034-1384587) and to increase the accuracy and power of the study, in this clinical trial, we plan to enroll 40 participants, which, with a 15% attrition rate, will increase to 46 subjects (23 in each group). Regarding the attrition rate, the dropout rate can be determined using historical data from similar studies or pilot studies. A common rule of thumb is to assume a 10-20% dropout rate (Patel et al, DOI:10.55489/njcm.150620243815). Studies conducted on melatonin supplementation in Iran (such as Mohammadi et al, https://doi.org/10.1155/2021/3502325, Mesri Alamdari et al, http://dx.doi.org/ 10.1055/s-0034-1384587, Jamilian et al, <https://doi.org/10.3389/fendo.2019.00273>, Shabani et al, <https://doi.org/10.1016/j.jad.2019.02.066>, Bahrami et al, <https://doi.org/10.1108/NFS-012019-0018>, Bahrami et al, <https://doi.org/10.1016/j.ctim.2020.102452>, Pakravan et al, https://doi.org/10.4103/2277-9175.204593) found a maximum rate of 12.5%. Therefore, we have decided to consider the attrition rate to be 15%.

-) While you have outlined a comprehensive set of inclusion and exclusion criteria, it’s still unclear how these have been verified during screening.

As mentioned in the lines 129-131, the present pilot study is the second phase of a Ph.D. dissertation. The first phase of the project is cross-sectional, and approximately 400 overweight or obese females will be recruited. The mentioned inclusion and exclusion criteria will be assessed in the first phase of the project (p.7, lines: 151-153).

-) The block size is mentioned as two. It might be helpful to confirm that this approach maintains the required balance across groups, as the small block size may not provide sufficient flexibility to ensure even distribution, particularly toward the end of the randomization process.

We will change the block size to four (p.8, lines 164-165).

-) It’s good that one author (RM) created the randomization sequence without being involved in assigning participants. This could be even stronger if it’s mentioned whether they are also blinded to any participant details or baseline data to reduce bias further.

We applied this suggestion (p.9, line 180).

-) Please specify the software used for randomization.

Randomization is conducted using the Random Allocation Software (RAS). It is mentioned in p.8, lines 163-164.

-) Specify the purpose of bi-weekly follow-up calls.

We changed this phrase to: weekly follow-up calls to improve response rates and fewer dropouts (p.9, lines 189-190).

-) Please clarify if there’s a standard method for counting and recording any unused tablets at each checkpoint to help ensure accurate tracking of compliance.

The total intake of tablets for each subject is calculated by subtracting the number of tablets unused from the number of tablets dispensed (p.9, lines 195-196).

With Regards,

Reza Mahdavi

Nutrition Research Center, Department of Biochemistry and Diet Therapy, Faculty of Nutrition and Food Sciences, Tabriz University of Medical Sciences, Tabriz, Iran

Email: [mahdavir@tbzmed.ac.ir](mailto:mahdavir@tbzmed.ac.ir)

Bahram Pourghassem Gargari

Nutrition Research Center, Department of Biochemistry and Diet Therapy, Faculty of Nutrition and Food Sciences, Tabriz University of Medical Sciences, Tabriz, Iran

Email: [pourghassemb@tbzmed.ac.ir](mailto:pourghassemb@tbzmed.ac.ir)
